# Recommendations for the management of ST-elevation myocardial infarction after reperfusion

**DOI:** 10.47487/apcyccv.v6i3.508

**Published:** 2025-09-24

**Authors:** Frank W. Britto

**Affiliations:** 1 Servicio de Cardiología Clínica, Instituto Nacional Cardiovascular - INCOR, EsSalud. Lima, Peru. Servicio de Cardiología Clínica Instituto Nacional Cardiovascular - INCOR, EsSalud Lima Peru

**Keywords:** Acute Coronary Syndromes, Myocardial Infarction, Heart Failure, Myocardial Revascularization, Síndrome Coronario Agudo, Infarto de Miocardio, Insuficiencia Cardiaca, Revascularización Miocárdica

## Abstract

The management of ST-segment elevation myocardial infarction (STEMI) after reperfusion requires critical decision-making in patients with multivessel disease (MVD), acute heart failure, and left ventricular (LV) thrombus. Complete revascularisation of non-culprit lesions ≥70% is recommended either during the index procedure or within the first 19 days, particularly in haemodynamically stable patients. Coronary artery bypass grafting is indicated for high-risk anatomy or complex lesions following successful percutaneous coronary intervention, where a hybrid approach may combine percutaneous intervention with surgery, and timing should be adjusted according to whether a stent was implanted. Post-infarction heart failure, which occurs in 28-31% of cases, requires urgent management; continuous monitoring and timely intervention can reduce complications. In patients with cardiogenic shock or mechanical complications, intra-aortic balloon pump support and inotropes may be necessary. Right ventricular infarction is managed with volume resuscitation, urgent revascularisation, and, when required, pharmacological support or pacing. LV thrombus, more frequent after anterior infarction with LVEF <50%, mandates early detection by echocardiography and/or tomography, and prompt anticoagulation, preferably with warfarin, with triple therapy considered in patients at high thrombotic risk. This manuscript outlines recommendations to optimise prognosis through early, evidence-based, and individualised interventions.

## RESUMEN

El manejo del infarto agudo de miocardio con elevación del ST tras la reperfusión implica decisiones clave en pacientes con enfermedad de múltiples vasos (EMV), falla cardiaca aguda y trombo en ventrículo izquierdo (VI). Se recomienda la revascularización completa de las lesiones no culpables ≥70% durante la intervención inicial o en los primeros 19 días, especialmente en pacientes estables. La cirugía de *bypass* está indicada en anatomías de alto riesgo o lesiones complejas tras intervención coronaria exitosa, en las que la estrategia híbrida combina intervención percutánea con cirugía, ajustando tiempos según si se implantó un *stent*. La falla cardiaca posinfarto es frecuente (28-31%) y requiere tratamiento urgente. El monitoreo continuo y la pronta intervención reducen complicaciones. En situaciones de *shock* o complicaciones mecánicas, puede ser necesario el uso de balón intraaórtico y soporte inotrópico. El infarto de ventrículo derecho se maneja con soporte de volumen, revascularización urgente y, si es necesario, apoyo farmacológico o marcapasos. El trombo en VI, más frecuente en infartos anteriores con FEVI <50%, requiere diagnóstico precoz con ecocardiografía y/o tomografía y anticoagulación temprana, preferentemente con warfarina, considerando triple terapia en casos de alto riesgo trombótico. Este manuscrito presenta recomendaciones que buscan optimizar el pronóstico mediante una intervención precoz y personalizada, basada en la evidencia más reciente.

## Introduction

In October 1961, Desmond Julian established the first Coronary Care Unit (CCU) at Sydney Hospital. In Peru, the first CCU was inaugurated on August 12, 1969, at Guillermo Almenara Hospital. In 2000, the European Society of Cardiology created the Working Group on Acute Cardiac Care, dedicated to defining the role of the cardiologist in the newly designated Cardiac Intensive Care Unit (formerly CCU). In 2011, the Working Group redefined the scope of acute cardiac care, extending from pre-hospital management through the first seven days of hospitalisation, and the Acute Cardiac Care Association was established.

In 2013, clinical cardiology services were established in Peru with the mission of providing acute cardiac care (or hospital cardiology). More than 50% of hospitalised patients suffer from ischaemic coronary heart disease in its various forms and clinical presentations. Among these, ST-elevation myocardial infarction (STEMI) is one of the most significant.

Much has been written about the management of patients with myocardial infarction in the pre-hospital phase and about reperfusion strategies, depending on the logistics of the network and the admitting hospital. By contrast, recommendations for in-hospital management after reperfusion are less well developed or are presented in a fragmented manner. This manuscript aims to provide a practical overview of post-reperfusion management in patients with STEMI, with a particular focus on multivessel disease (MVD), acute post-infarction heart failure, and left ventricular (LV) thrombus.

## Management of uncomplicated STEMI and MVD

MVD refers to the presence of coronary lesions in vessels other than the culprit lesion (non-culprit lesion [NCL]). It is present in approximately 50% of patients with STEMI and is associated with increased 30-day mortality. ^1^

The 2025 Australian clinical guideline for the diagnosis and management of acute coronary syndromes ^2^ recommends complete revascularization of NCLs ≥70% (≥2.25 mm) (strong recommendation, high level of evidence) either during the index percutaneous coronary intervention (PCI) or within 19 days of the infarction (weak recommendation, moderate level of evidence). In the 2025 American College of Cardiology and partner society guideline for the management of acute coronary syndromes, ^3^ the recommendations are as follows:

• In haemodynamically stable patients with STEMI and MVD, after successful PCI of the infarct-related artery (IRA), PCI of NCL is recommended to reduce the risk of death or myocardial infarction and to improve quality of life (class I recommendation, level of evidence A).

• In patients with STEMI and MVD, following successful PCI of the IRA, coronary artery bypass grafting (CABG) is a reasonable option for high-risk anatomy (severe left main coronary artery [LMCA] stenosis, left main equivalent, or critical proximal left anterior descending [LAD] lesion) to reduce cardiovascular events (class IIa recommendation, level of evidence C).

• In selected patients with STEMI and low-complexity MVD, multivessel PCI during the index primary PCI (provided that IRA PCI is successful) is preferable over staged PCI (class IIb recommendation, level of evidence B). The MULTISTARS AMI ^4^^)^ trial evaluated the optimal timing of complete revascularisation. The immediate multivessel PCI strategy was superior to staged PCI beyond 19 days (Hazard Ratio [HR]: 0.52, 95% confidence Interval [CI]: 0.38-0.72; p<0.001). Notably, the benefit was driven by a reduction in non-fatal myocardial infarction, particularly periprocedural infarction, which is difficult to distinguish from the index event within the first 10 days.

These recommendations also apply to patients managed with a pharmacoinvasive strategy. ^(^^5^

### In-hospital care (Figure 1)

**Figure 1:**
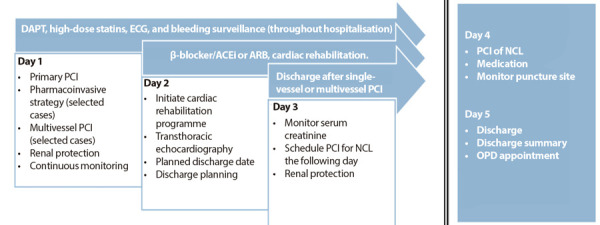
Clinical pathway for the management of patients with STEMI and multivessel disease.

DAPT: dual antiplatelet therapy. ECG: electrocardiogram. ACEI: angiotensin-converting enzyme inhibitor. ARB: angiotensin II receptor blocker. PCI: percutaneous coronary intervention. NCL: non-culprit lesions. OPD: outpatient department.

- After successful PCI of the IRA and in the presence of focal NCL ≥70%, multivessel PCI should be performed.

- Deferral of PCI for NCL is recommended if the patient presents with haemodynamic instability without hypoperfusion, angiographic features suggestive of unstable lesions, severe LMCA stenosis, LMCA equivalent, proximal LAD lesion, or serum creatinine >1.2 mg/dL. ^6^

- Implement periprocedural renal protection.

- Administer dual antiplatelet therapy (DAPT): aspirin plus ticagrelor or clopidogrel.

- Administer atorvastatin (40-80 mg) or rosuvastatin (20-40 mg) if transaminase levels are less than ten times the upper limit of normal (if greater than ten times, defer therapy until levels decline and active liver disease can be excluded). ^(^^7^

- If revascularisation of NCL was not performed during primary PCI, wait 48-72 h, measure serum creatinine, and if within normal limits, schedule PCI of NCL ≥70% (with renal protection). After PCI, inspect the puncture site, obtain an electrocardiogram, and if no complications are detected, discharge can be considered the following day.

- Provide continuous monitoring (including serial electrocardiograms) and surveillance for bleeding in an intensive care unit or equivalent setting, throughout hospitalisation.

- In single-vessel cases, patients can be transferred back to their referring hospital if treated in a hub centre (“repatriation”).

- Initiate a β-blocker ^3^ and/or an angiotensin-converting enzyme (ACE) inhibitor or angiotensin II receptor blocker (ARB). The use of β-blockers in patients with preserved left ventricular ejection fraction (LVEF) after STEMI has recently been questioned. ^8^^,^^9^ Differences in effectiveness across meta-analyses appear to reflect variations in design, methodology, and statistical analysis. The wide divergence in guideline recommendations adds to the uncertainty. Pending results of ongoing trials, β-blockers should continue to be prescribed in the absence of contraindications. ^3^

- Perform transthoracic echocardiography promptly to assess LVEF, wall motion score index, and E/e’.

- Initiate cardiac rehabilitation. ^(^^10^

- Define the expected date of discharge (assuming no clinical, infrastructural, equipment, supply, or administrative complications), ^11^ and organise a comprehensive discharge plan (addressing both cardiovascular and non-cardiovascular needs). ^2^^,^^11^ Additionally, provide a discharge summary ^12^^,^^13^^)^ documenting the type of reperfusion, timings, procedures performed, LVEF, LDL cholesterol, HbA1c, and the proposed duration of DAPT.

### What about late comers?

Patients who present between 12 h and 48 h after symptoms onset to a centre with reperfusion capability (pharmacological or mechanical) are referred to as late comers. ^14^^,^^15^ Those who are asymptomatic and haemodynamically stable should be referred for coronary angiography (class IIa recommendation, level of evidence B). ^3^^,^^16^

In such cases, the following is recommended:

• Percutaneous revascularisation of the IRA within 48 h of the event, irrespective of the initial Thrombolysis In Myocardial Infarction (TIMI) flow. In patients with high-risk anatomy (LMCA stenosis or proximal LAD lesion), surgical myocardial revascularisation should be performed.

• For patients presenting more than 48 h after symptoms onset:

- IRA with TIMI III flow: perform PCI.

- IRA with TIMI 0 flow and non-high-risk anatomy: discharge may be considered, with an ischaemia-inducing test (ideally imaging-based) scheduled at 3 months. ^(^^17^

### Coronary artery bypass grafting of NCL after PCI of the IRA

We do not have registries or controlled trials that clearly define the optimal strategy after successful revascularisation of the IRA. Current practice is guided by expert opinion, which suggests considering CABG of NCL, typically when the IRA is the right coronary artery, in the following scenarios ^18^: high-risk anatomy (LMCA stenosis, LMCA equivalent, or proximal LAD lesion), high anatomical complexity (SYNTAX score ≥22), LVEF ≤35% with critical lesions in both the LAD and circumflex arteries, diabetes mellitus, the presence of chronic total occlusion, and/or high bleeding risk coexisting with high ischaemic risk.

In these cases, the preoperative recommendations are as follows:

• Optimal timing of surgery.^19^

- If TIMI III flow was achieved in the IRA and no stent was implanted, parenteral anticoagulation plus aspirin should be continued, while the P2Y12 receptor inhibitor should be withheld. CABG should be performed between the fourth and seventh day. 

- If a stent was implanted in the IRA, DAPT should be continued until day three, the P2Y12 receptor inhibitor withheld on day four, and CABG performed between days 7 and 9.

• Regarding parenteral anticoagulation, unfractionated heparin should be maintained until surgery ^3^, whereas low molecular weight heparin should be discontinued 18-24 h before CABG.

• Red blood cell transfusion should not be used solely to “optimise” haemoglobin, as it may promote stent thrombosis (due to interruption of DAPT, multiple stents, and increased platelet reactivity related to alterations in the adenosine diphosphate [ADP] pathway in transfused blood). ^20^

## Post-infarction heart failure

In the PERSTEMI II registry, ^21^ the incidence of post-infarction heart failure (PIHF) was 27.8% (excluding cases of cardiogenic shock [11.5%]). In the INCOR STEMI registry, for 2024 (Pitta M, *et al*., unpublished data), the incidence was 30.8%, making it the most frequent complication. Between 10% and 15% of these patients will die or be hospitalised for heart failure (HF) within the following 2 years. ^22^^,^^23^

Both Gheorghiades M. ^24^ and the European statement on acute HF and acute coronary syndromes ^25^ emphasise that the specific treatment for STEMI complicated by HF is emergency coronary angiography and immediate revascularisation. But what about periprocedural management and the approach to patients who fail to stabilise promptly? This will be addressed below, as the European guidelines on acute coronary syndromes ^16^ and those from the American College of Cardiology/American Heart Association ^3^ are either brief on this subject or refer readers to documents focused on the use of diuretics in patients with congestion. ^26^

Ferrero *et al*. ^27^ compared mortality according to the Killip-Kimball (KK) classification over more than 50 years of technological advances (1967-2021) (Table 1), showing that despite its age, it remains a valuable tool for predicting mortality at admission and, therefore, guiding treatment decisions.

**Table 1:** In-hospital mortality according to Killip-Kimball classification

Killip-Kimball class	In-hospital mortality (%)		
	Ferrero (2021)	Original (1967)
I	3.5	6
II	6.9	17
III	25	38
IV	57	81

The clinical presentation generally includes orthopnoea, tachycardia, absence of hypotension, tachypnoea, arterial oxygen saturation <95%, and pulmonary crackles. Chest radiography typically ranges from pulmonary hyperflow to pulmonary oedema. Bedside lung ultrasound is increasingly used to confirm pulmonary congestion and detect subclinical congestion; however, therapeutic targets have not yet been established for this subgroup (KK I with subclinical congestion). ^28^

### Pathophysiology of post-infarction heart failure

The Canadian Cardiovascular Society and the Canadian Heart Failure Society define five phenotypes of decompensated heart failure: hypertensive acute pulmonary oedema, acute or *de novo* HF, worsening of chronic HF, the cardiorenal syndrome, and frailty and/or hypotension. ^29^ Post-infarction HF corresponds to acute or *de novo* HF, in which two distinct but temporally sequential pathophysiological mechanisms are involved. ^30^

- Acute mechanisms (first activated): myocardial loss triggers rapid sympathetic activation, leading to increased afterload and mobilisation of blood from the splanchnic reservoir into the circulating volume. This results in increased preload, which precipitates pulmonary congestion.

- Subacute mechanisms (days to weeks): activation of the renin-angiotensin-aldosterone system due to reduced renal perfusion promotes sodium and water retention, sustaining pulmonary congestion and subsequently systemic congestion.

Under normal conditions, pulmonary alveolar epithelium continuously generates interstitial fluid, the clearance of which is regulated by active sodium and chloride transport. ^31^ Sodium enters the alveolar epithelial cell through a specific channel, creating an inward chloride current to maintain electroneutrality. Sodium is then exchanged for potassium via the Na-K ATPase pump and transported to the interstitium, followed by water. ^32^ This process is facilitated by nitric oxide released from pulmonary capillary endothelium in response to elevated hydrostatic pressure. ^33^ In pulmonary oedema, sodium channel activity is inhibited, generating a gradient that activates the NaK2Cl channel. Sodium and chloride exit into the interstitium, producing an electromotive gradient that drives active fluid secretion into the interstitial space. Resolution of pulmonary oedema depends on active sodium reabsorption and intact lymphatic drainage capacity to remove fluid from the alveolar space. In animal models, loop diuretics have been shown to reduce chloride flux, whereas spironolactone enhances sodium channel activity and accelerates alveolar fluid clearance.

### Management recommendations for PIHF after successful PCI or when systolic blood pressure (SBP) is greater than 90 mmHg (Figure 2)

**Figure 2:**
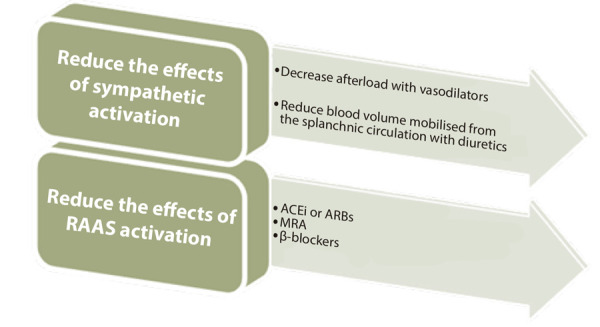
Pathophysiology-based treatment of post-infarction heart failure.

RAAS: renin-angiotensin-aldosterone system. MRA: mineralocorticoid receptor antagonist. ACEi: angiotensinconvertingenzyme inhibitors. ARB: angiotensin II receptor blocker.

• Semi-upright positioning, frequent monitoring, and oxygen therapy if needed to maintain arterial oxygen saturation >95%.

• Administer intravenous (IV) furosemide 20-40 mg as a bolus if systolic blood pressure (SBP) is >90 mmHg (aiming for venodilatation and reduction of mean right atrial pressure). ^27^^,^^34^^-^^36^

• Intravenous nitroglycerin (NTG) infusion if SBP ≥100 mmHg ^37^ to reduce afterload and splanchnic volume redistribution.

• Patients with PIHF are at least at moderate risk of contrast-induced nephropathy. Conventional protocols cannot be applied; therefore, the POSEIDON study protocol is suggested, consisting of hydration with normal saline at 3 mL/kg for 1 h, followed by 1.5 mL/kg/h for 4 h. ^(^^38^^-^^40^

• Maintain serum potassium between 4.0 and 5.5 mEq/L. ^(^^41^

• From day 2, initiate an ACE inhibitor or ARB and taper NTG. In addition, administer a mineralocorticoid receptor antagonist if indicated. ^42^^-^^44^

• Management recommendations for PIHF after unsuccessful PCI or when SBP is below 90 mmHg. ^45^

• Perform echocardiography to rule out mechanical complications.

• Review coronary anatomy and assess the need to return to the catheterisation laboratory.

• Insert an arterial line and central venous catheter; consider placement of a pulmonary artery catheter.

• Implant an intra-aortic balloon pump (IABP) 1:1 for 24 h. ^45^ Subsequently, if SBP is ≥100 mmHg (without IABP support for 3 min), urine output is >100 mL/h during the previous 3 h, and clinical improvement of congestion is evident, switch to 2:1 support and discontinue heparin for 2 h. Then measure aPTT: if ≤40 s, remove the balloon; if >40 s, wait an additional 2 h before removal. Conversely, if SBP remains <90 mmHg and/or urine output <90 mL in the last 3 h and/or pulmonary congestion persists, maintain 1:1 support for a further 12 h.

• Inotropes:

- Levosimendan ^46^: loading dose of 6-12 µg/kg over 10 min (in patients on IABP or vasopressors), followed by IV infusion at 0.1-0.2 µg/kg/min.

- Dobutamine ^(^^47^: although randomised studies in acute myocardial infarction are lacking, it is known to increase cardiac output and reduce left ventricular end-diastolic pressure without enlarging infarct size, provided dosing does not raise heart rate by >10% above baseline and/or >90 mmHg.

### Post-infarction heart failure due to right ventricular (RV) infarction

In 1930, Saunders described a clinical triad in a patient with extensive RV necrosis: arterial hypotension, jugular venous distension, and clear lung fields. ^48^ RV infarction typically occurs in association with an inferoposterior infarction, with the right coronary artery almost always being the IRA. Most patients experience early haemodynamic improvement and recovery of RV function, even in the absence of reperfusion. ^49^

The pathophysiology involves an acute reduction in RV contractility, leading to a marked decrease in LV preload, reduced cardiac output, and arterial hypotension. Additionally, RV diastolic dysfunction develops, which increases right atrial (RA) pressure, making RV filling dependent on right atrial contraction. Haemodynamic deterioration is therefore more pronounced when atrioventricular synchrony is lost. ^50^

RV infarction should be suspected in any patient with an inferior myocardial infarction (hence the importance of obtaining right-sided ECG leads), in cases of sinus bradycardia with or without hypotension and jugular venous distension, or in patients who develop hypotension after nitroglycerin administration. Echocardiography typically demonstrates reduced tricuspid annular plane systolic excursion (TAPSE), decreased RV fractional shortening, and RV dilatation.

Management, in addition to cardiac catheterisation and emergency revascularisation, includes:

- In the presence of hypotension, discontinue NTG and administer 250 mL of normal saline over 30 min.

- If hypotension persists, insert a central venous catheter to measure RA pressure. ^51^ If RA pressure is <10 mmHg, increase it to 12-14 mmHg with normal saline at 40 mL/min (up to a maximum of 2 L). If SBP does not rise above 90 mmHg, stop fluid administration and initiate norepinephrine to achieve an SBP of 90 mmHg, in addition to dobutamine. Note that RA pressure measurements are unreliable in the presence of high-grade atrioventricular (AV) block due to AV dissociation.

- In a complete AV block, administer intravenous atropine 1 mg as a bolus, repeated 2-3 times at 15-minute intervals. If sinus rhythm or first-degree AV block is not restored, implant a transvenous pacemaker according to SBP: if SBP <90 mmHg, use a dual-chamber device with an AV delay of 100-150 ms, adjusted by response; ^52^ if SBP ≥90 mmHg, use a single-chamber pacemaker.

## Management of patients with left ventricular thrombus

In the pre-thrombolytic era, the incidence of LV thrombus was approximately 60%, particularly in anterior myocardial infarctions. ^53^ With thrombolysis, the incidence declined to below 40%, ^54^ and with primary PCI and the use of cardiac magnetic resonance (CMR) for LV thrombus detection, it has fallen to around 19%, especially in anterior infarctions with LVEF <50%. ^55^ Most LV thrombi develop within the first 14 days. ^56^

CMR is the best imaging modality for the diagnosis of LV thrombus, but its limited availability and high cost are major barriers. Thus, a combination of transthoracic echocardiography (TTE) and cardiac CT may guide management. ^56^^,^^57^ It is suggested to perform TTE within the first 48 h; ^57^ if no thrombus is detected but suspicion remains, contrast-enhanced cardiac CT or contrast echocardiography at day 5 may be useful, reserving CMR for complex or equivocal cases. ^57^

If LV thrombus is confirmed, anticoagulation with warfarin is recommended (low-dose rivaroxaban may be considered if warfarin is not tolerated) ^58^, according to the following scheme:

• Continue parenteral anticoagulation plus DAPT and initiate warfarin to achieve an INR of 2.0-2.5; maintain DAPT and warfarin for 7 days, then continue warfarin plus a P2Y12 inhibitor for a total of 3 months. In patients at high thrombotic risk, triple therapy should be maintained for 1 month. ^59^

• In the absence of contrast-enhanced TTE, cardiac CT, or CMR, triple therapy should be initiated as above in patients with the following characteristics: QS waves in V1-V4 on ECG, cumulative ST elevation >10 mm, or LVEF <40%. ^60^^,^^61^
